# Supplementary material for: QuanTI-FRET: a framework for quantitative FRET measurements in living cells
Source: Sci Rep. 2020 Apr 16;10:6504. doi: 10.1038/s41598-020-62924-w (PMC7162988; doi:10.1038/s41598-020-62924-w)
Supplement: Supplementary file 1 — Supplementary information. [file 41598_2020_62924_MOESM1_ESM.pdf]

QuanTI-FRET: a framework for quantitative FRET measurements in living cells

Alexis Coullomb[1], Cécile M. Bidan[1], Chen Qian[2], Fabian Wehnekamp[2], Christiane Oddou[3], Corinne Albigès-Rizo[3], Don. C. Lamb[2], and Aurélie Dupont[1,*]

[1]Univ. Grenoble Alpes, CNRS, LIPhy, Grenoble, F-38000, France
[2]Department of Chemistry, Center for Nano Science (CENS), Center for Integrated Protein Science (CIPSM) and Nanosystems Initiative München (NIM), Ludwig Maximilians-Universität München, Germany
[3]Institute for Advanced Biosciences, Université Grenoble Alpes, INSERM U1209, UMR5309, F38700 La Tronche, France
[*]aurelie.dupont@univ-grenoble-alpes.fr

Contents

Derivation of the FRET efficiency equation from the fluorescence images 2
Effect of the addition of free donor 3
Distribution of the crosstalk correction factors 4
Detailed analysis of three exemplary cells 5
Cellwise representation of FRET probability with respect to the fluorescence intensity 6
Stoichiometry distributions for the different FRET standards measured 7
Anomalous FRET standard, CTV 8
Comparison of the QuanTI-FRET robustness with two other published methods 9
Comparative table gathering the calibration and results from the two independent laboratories 10
References 10

Derivation of the FRET efficiency equation from the fluorescenece images

In the main text, we wrote the fluorescence intensity in the three collected images as a function of the photophysical and instrumental parameters, the number of donor, n^D, and acceptor, n^A , fluorophores in the considered pixel and FRET probability, E:

$$I_{AA} = n^A L_A \sigma^A_{Aex} \phi_A \eta^{Aem}_{Adet} \quad (1)$$

$$I_{DD} = n^D L_D \sigma^D_{Dex} (1-E) \phi_D \eta^{Dem}_{Ddet} \quad (2)$$

$$I_{DA} = n^D L_D \sigma^D_{Dex} E \phi_A \eta^{Aem}_{Adet} + n^D L_D \sigma^D_{Dex} (1-E) \phi_D \eta^{Dem}_{Adet} + n^A L_D \sigma^A_{Dex} \phi_A \eta^{Aem}_{Adet} \quad (3)$$

We can express I_{DA} as a function of I_{AA} and I_{DD} by replacing n_D and n_A using equations 1 and 2:

$$I_{DA} = \frac{\phi_A \eta^{Aem}_{Adet}}{\phi_D \eta^{Dem}_{Ddet}} \frac{E}{1-E} I_{DD} + \frac{\eta^{Dem}_{Adet}}{\eta^{Dem}_{Ddet}} I_{DD} + \frac{L_D \sigma^A_{Dex}}{L_A \sigma^A_{Aex}} I_{AA} \quad (4)$$

We then defined correction factors so as to simplify the expressions:

$$\alpha^{BT} = \frac{\eta^{Dem}_{Adet}}{\eta^{Dem}_{Ddet}} \quad \delta^{DE} = \frac{L_D \sigma^A_{Dex}}{L_A \sigma^A_{Aex}} \quad \gamma^M = \frac{\phi_A \eta^{Aem}_{Adet}}{\phi_D \eta^{Dem}_{Ddet}} \quad \text{and} \quad \beta^X = \frac{L_A \sigma^A_{Aex}}{L_D \sigma^D_{Dex}} \quad (5)$$

Replacing in equation 4, it comes:

$$I_{DA} = \gamma^M \frac{E}{1-E} I_{DD} + \alpha^{BT} I_{DD} + \delta^{DE} I_{AA} \quad (6)$$

We can rearrange this last equation to obtain E:

$$E = \frac{I_{DA} - \alpha^{BT} I_{DD} - \delta^{DE} I_{AA}}{I_{DA} - \alpha^{BT} I_{DD} - \delta^{DE} I_{AA} + \gamma^M I_{DD}} \quad (7)$$

This is equation (5) in the main text. To obtain the stoichiometry as defined by equation (6) in the main text, we replace n^A and n^D by their expression obtained from equations 1 and 2:

$$S = \frac{1}{1 + \frac{n^A}{n^D}} = \frac{1}{1 + \frac{1}{\gamma^X \gamma^M} \frac{I_{AA}}{I_{DD}} (1-E)} \quad (8)$$

Next, replacing E by its expression (equation 7), the stoichiometry reads:

$$S = \frac{I_{DA} - \alpha^{BT} I_{DD} - \delta^{DE} I_{AA} + \gamma^M I_{DD}}{I_{DA} - \alpha^{BT} I_{DD} - \delta^{DE} I_{AA} + \gamma^M I_{DD} + I_{AA}/\beta^X} \quad (9)$$

Finally, we further simplify the notation by defining $I^{corr}_{DA} = I_{DA} - \alpha^{BT} I_{DD} - \delta^{DE} I_{AA}$. Thus, we obtain two master equations defining the FRET probability and the stoichiometry in each pixel:

$$E = \frac{I^{corr}_{DA}}{I^{corr}_{DA} + \gamma^M I_{DD}} \quad (10)$$

$$S = \frac{I^{corr}_{DA} + \gamma^M I_{DD}}{I^{corr}_{DA} + \gamma^M I_{DD} + I_{AA}/\beta^X} \quad (11)$$

Effect of the addition of free donor

Here, we describe theoretically the effect of a deviation from the expected stoichiometry on the E-S 2D-histogram. We consider a construct with a FRET efficiency E_0 in quantity n_0^D $(= n^A)$, we write the intensities equations as described in the main text:

$$I_{AA} = n^A L_A \sigma^A_{Aex} \phi_A \eta^{Aem}_{Adet} \tag{12}$$
$$I^0_{DD} = n^D_0 L_D \sigma^D_{Dex} (1 - E_0) \phi_D \eta^{Dem}_{Ddet} \tag{13}$$
$$I^0_{DA} = L_D \phi_A \eta^{Aem}_{Adet} (n^D_0 \sigma^D_{Dex} E_0 + n^A \sigma^A_{Dex}) + n^D_0 L_D \sigma^D_{Dex} \phi_D \eta^{Dem}_{Adet} (1 - E_0) \tag{14}$$

We add to this population a free donor species in the quantity of n_D^{free} which adds to the intensities:

$$I^{free}_{DD} = n^D_{free} L_D \sigma^D_{Dex} \phi_D \eta^{Dem}_{Ddet} \tag{15}$$
$$I^{free}_{DA} = n^D_{free} L_D \sigma^D_{Dex} \phi_D \eta^{Dem}_{Adet} \tag{16}$$

In total, the measured intensities in the three channels will be:

$$I_{AA} = n^A L_A \sigma^A_{Aex} \phi_A \eta^{Aem}_{Adet} \tag{17}$$
$$I_{DD} = L_D \sigma^D_{Dex} \phi_D \eta^{Dem}_{Ddet} [n^D_0 (1 - E_0) + n^D_{free}] \tag{18}$$
$$I_{DA} = L_D \phi_A \eta^{Aem}_{Adet} (n^D_0 \sigma^D_{Dex} E_0 + n^A \sigma^A_{Dex}) + L_D \sigma^D_{Dex} \phi_D \eta^{Dem}_{Adet} [n^D_0 (1 - E_0) + n^D_{free}] \tag{19}$$

Now, replacing I_{AA}, I_{DD} and I_{DA} in the FRET equation (9) in the main text:

$$E = \frac{I_{DA} - \alpha^{BT} I_{DD} - \delta^{DE} I_{AA}}{I_{DA} - \alpha^{BT} I_{DD} - \delta^{DE} I_{AA} + \gamma^M I_{DD}}, \tag{20}$$

it becomes after some rearrangements:

$$E_{app} = \frac{1}{1 + \frac{1 - E_0 + n^D_{free}/n^D_0}{E_0}} \tag{21}$$

From the definition of S (Eq.10 , main text), we obtain:

$$S_{app} = \frac{1 + n^D_{free}/n^D_0}{1 + n^D_{free}/n^D_0 + n^A/n^D_0} = \frac{1 + n^D_{free}/n^D_0}{1/S_0 + n^D_{free}/n^D_0}. \tag{22}$$

By combining the equations of E_{app} and S_{app} and eliminating n^D_{free}/n^D_0, it finally becomes:

$$S_{app} = \frac{E_0/E_{app}}{1/S_0 + E_0/E_{app} - 1} \tag{23}$$

This is the equation of the line sketched in figure 3. The addition of free acceptors to the same "ideal" sample (FRET efficiency E_0 in quantity n_0^D and $= n_0^A$) has no effect on the FRET probability; only the stoichiometry is affected and decreases.

Distribution of the crosstalk correction factors

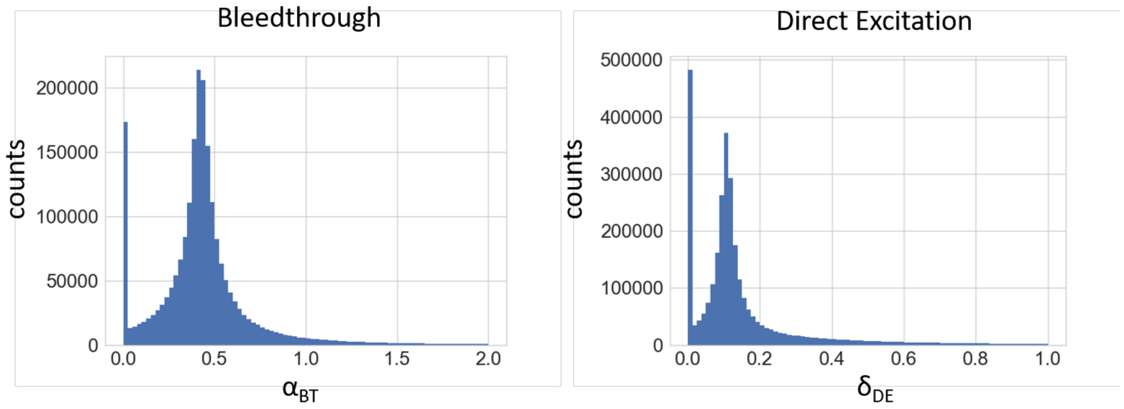

Figure S1: Distributions of α^{BT} and δ^{DE} calculated on every single pixel of the donor-only cells (α^{BT}) and acceptor-only cells (δ^{DE}). A well-defined peak is visible in both cases.

Living cells expressing either Cerulean or Venus were imaged and an automatic threshold was applied to keep only pixels from within the cells (Huang method in ImageJ). The bleedthrough correction factor α^{BT} was calculated as the ratio between the FRET channel and the Donor channel, I_{DA}/I_{DD}, in cells expressing only the donor fluorophore (10 cells) for all pixels above the threshold. The pixelwise distribution is shown in Fig.S1 (left panel) resulting in a median value $\alpha^{BT} = 0.4214$. Since the distribution is not gaussian, we estimated the confidence interval (CI_{68}) equivalent to 1σ in a gaussian distribution by simply calculating the interval containing 68% of the values around the median, $\alpha^{BT} = 0.4214 \pm 0.184$. The confidence on the determination of the peak was estimated similarly to the standard error of the mean, dividing by the square root of the sample size. But, all the measured pixels are not independent. We took into account the size of the PSF which includes $n_{PSF} = 15$ pixels and the number of imaged cells ($N_{cell} = 10$). The number of independent values was therefore estimated as the total number of pixels ($N = 2.1 \cdot 10^6$) normalized by the PSF and the number of independent images: $N_{eff} = N/(n_{PSF} \cdot N_{cell}) = 1.45 \cdot 10^4$. Finally, we estimated the error on the α^{BT} determination to be: $CI_{68}/\sqrt{N_{eff}} = 2 \cdot 10^{-3}$.

Identically, fluorescence images of cells expressing only the acceptor (12 cells) were taken, automatically thresholded resulting in the pixelwise distribution shown in Fig.S1 (right panel). The median value was $\delta^{DE} = 0.1100$ with a confidence interval $CI_{68} = 0.1100$. Through the same procedure as for α^{BT}, the uncertainty on the δ^{DE} determination was $CI_{68}/\sqrt{N_{eff}} = 8 \cdot 10^{-4}$ ($N = 3.0 \cdot 10^6$).

Detailed analysis of three exemplary cells

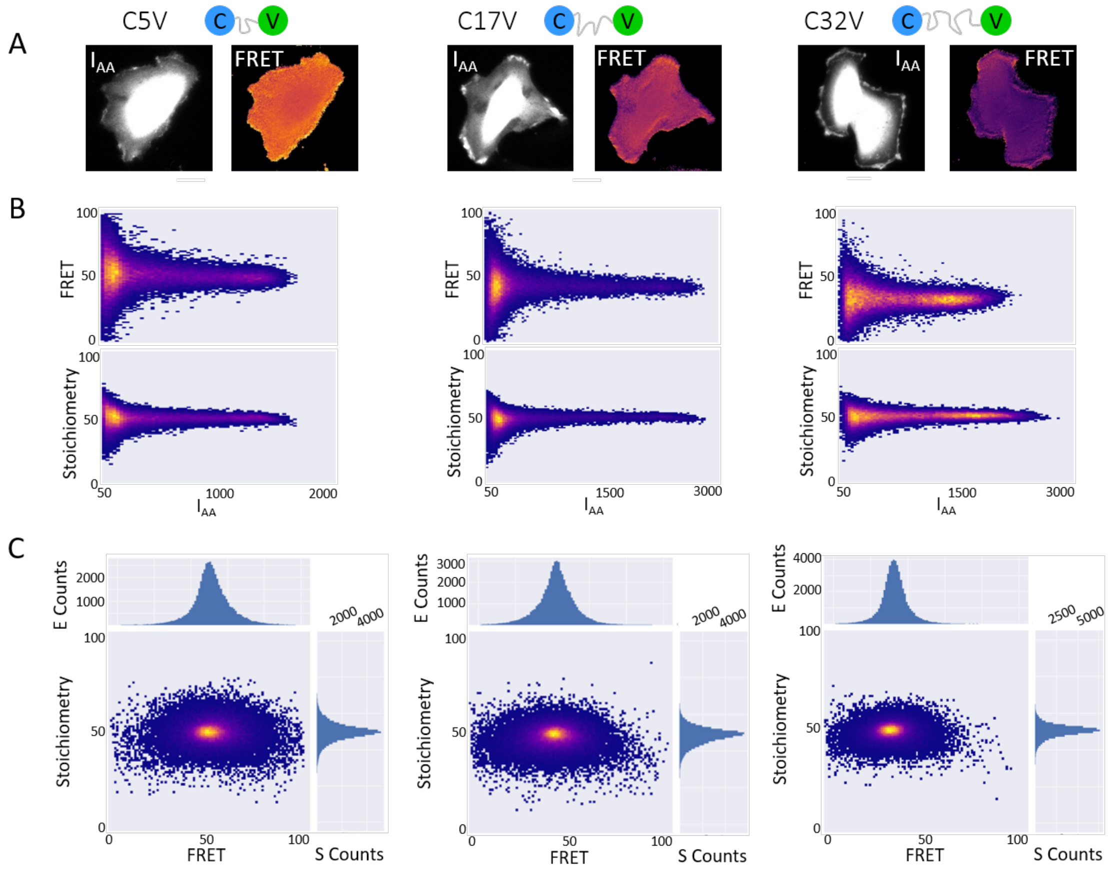

Figure S2: (A) Three exemplary cells from Figure 2. (B) FRET and stoichiometry histograms as a function of the pixel intensity, no correlation is visible. (C) 2D E-S histograms showing sharp peaks centered around the 50% stoichiometry.

We utilized the FRET standards developed by Thaler et al.[1] and Koushik et al.[2], they consist of a pair of fluorescent proteins, a donor (Cerulean) and an acceptor (Venus), separated by an amino-acid sequence of variable length. Here, we show a detailed analysis of the three exemplary cells presented in the main text (Fig.2), they express C5V, C17V or C32V, where the linker between donor and acceptor consisted respectively of 5, 17 and 32 amino-acids. The FRET probabilities and stoichiometries are shown as 2D histograms (pixelwise) as a function of the fluorescence intensity in channel I_{AA} (panel B). The intensity I_{AA} is a direct indicator of the acceptor fluorophore concentration. The FRET probability and the stoichiometry are expected to be independent of I_{AA} if the experimental system is perfectly calibrated. Visually, this can be verified for these three cells in Supplementary Fig.S2, panel B. The global correlation coefficients were calculated in the main text and confirmed that with QuanTI-FRET the FRET probability and the stoichiometry become independent of the fluorescence intensity. As supplementary information in panel C, we show the E-S 2D-histogram for each cell. A clear peak is visible for a stoichiometry about 50% and a particular FRET probability corresponding to the specific construct.

Cellwise representation of FRET probability with respect to the fluorescence intensity

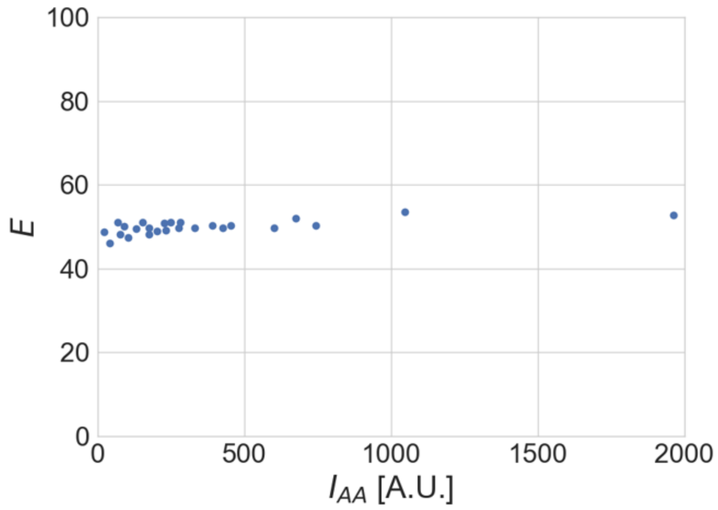

Figure S3: Cellwise median FRET efficiency as a function of the median intensity in the third channel (I_{AA}), each point represents a cell expressing the C5V construct.

Another way to show the relation between FRET probability and fluorescence intensity is to plot the results cellwise. Figure S3 shows the median FRET probability versus the median fluorescence intensity in channel I_{AA} for the different cells measured. Here again , no clear correlation is observable.

Stoichiometry distributions for the different FRET standards measured

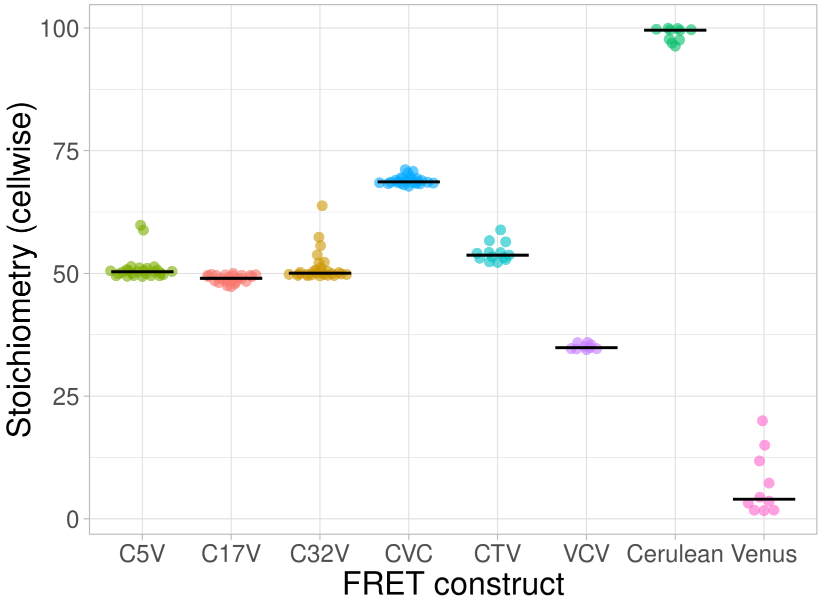

Figure S4: Stoichiometry distributions for the different FRET standards measured. Each point represents the median stoichiometry from one cell, the median of these cell median values is noted with a horizontal line.

Here are shown the cellwise distributions of the stoichiometry for all the measured constructs. Except for a few aberrant cells, the three FRET standards used for calibration (C5V, C17V and C32V) show cell median values very close to 50% as defined by the calibration. Two constructs with different stoichiometries were tested: CVC (2 donors: 1 acceptor) and VCV (1 donor : 2 acceptors). The median stoichiometries measured per cell were on average $S = 68.9 \pm 0.2$ (mean $\pm$ s.e.m) for CVC (24 cells with an expected value of 66%), and $S = 35.1 \pm 0.2$ for VCV (9 cells with an expected value of 33%). Even if all the calculation is based on the FRET phenomenon, it is still possible to calculate the stoichiometry for pure donor (Cerulean) and pure acceptor (Venus) samples. From the donor-only cells, the stoichiometry was calculated to be $S = 98.7 \pm 0.4$ (10 cells), close to the expected value of 100%. The distribution of stoichiometries for the acceptor-only cells is broader ($S = 7 \pm 2$) with some cells showing stoichiometries up to 20%. This is related to the very low signal-to-noise ratio for those cells yielding a close-to-zero value in the denominator for the calculation of S, the numerator being also close to zero for acceptor-only cells. For CTV, the stoichiometry is always larger than 50%. This is discussed in the next section.

Anomalous FRET standard, CTV

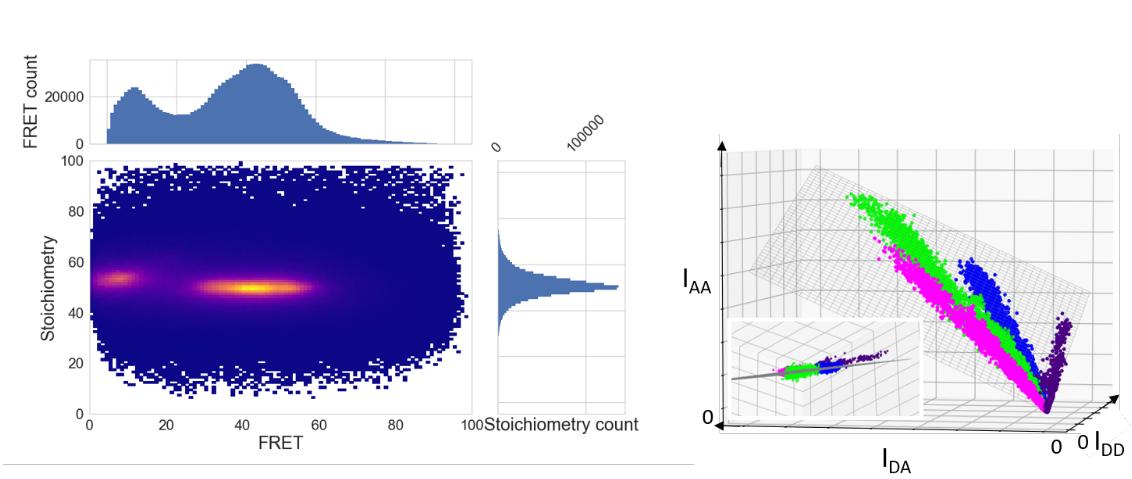

Figure S5: *Left* An E-S 2D histogram presenting CTV, C5V, C17V and C32V constructs. While the C5V, C17V and C32V clouds overlap and are flat with stoichiometries of $\simeq 0.5$, the CTV cloud is at very low FRET values but tilted and slightly above S=0.5. *Right* A 3D plot representing the 3-channel acquisition of CTV (indigo), C5V (blue), C17V (lime) and C32V (magenta). The CTV cloud is off the fitted plane.

In their article introducing different FRET standards with varying donor:acceptor stoichiometries, Thaler et al.[1] present the so-called CTV construct where the donor and acceptor fluorophores were separated by the TRAF domain of human TRAF2. This spacer is 229 amino-acid long and the crystal structure predicts at least a 80Å between Cerulean and Venus[1]. Cells expressing the CTV construct were measured with the QuanTI-FRET method. The E-S 2D histogram gathering the results for CTV as well as for C5V, C17V and C32V is shown in Fig.S5, left panel. The CTV population is visible at very low FRET efficiencies as expected but the cloud is tilted denoting a correlation between E and S. By looking into the 3D representation presented in the main text, we observe that the pixels representing CTV fluorescence (indigo) do not lie in the same plane as the other constructs (Fig.S5, rigth panel). This discrepancy can originate from the tendancy of the TRAF2 domain to form trimers thereby bringing the fluorophores in close contact causing energy transfer from Cerulean to several Venus acceptors[3]. As a consequence, CTV was not used to calibrate the setups as in the work by Chen et al.[4] and should be avoided for this purpose.

Comparison of the QuanTI-FRET robustness with two other published methods

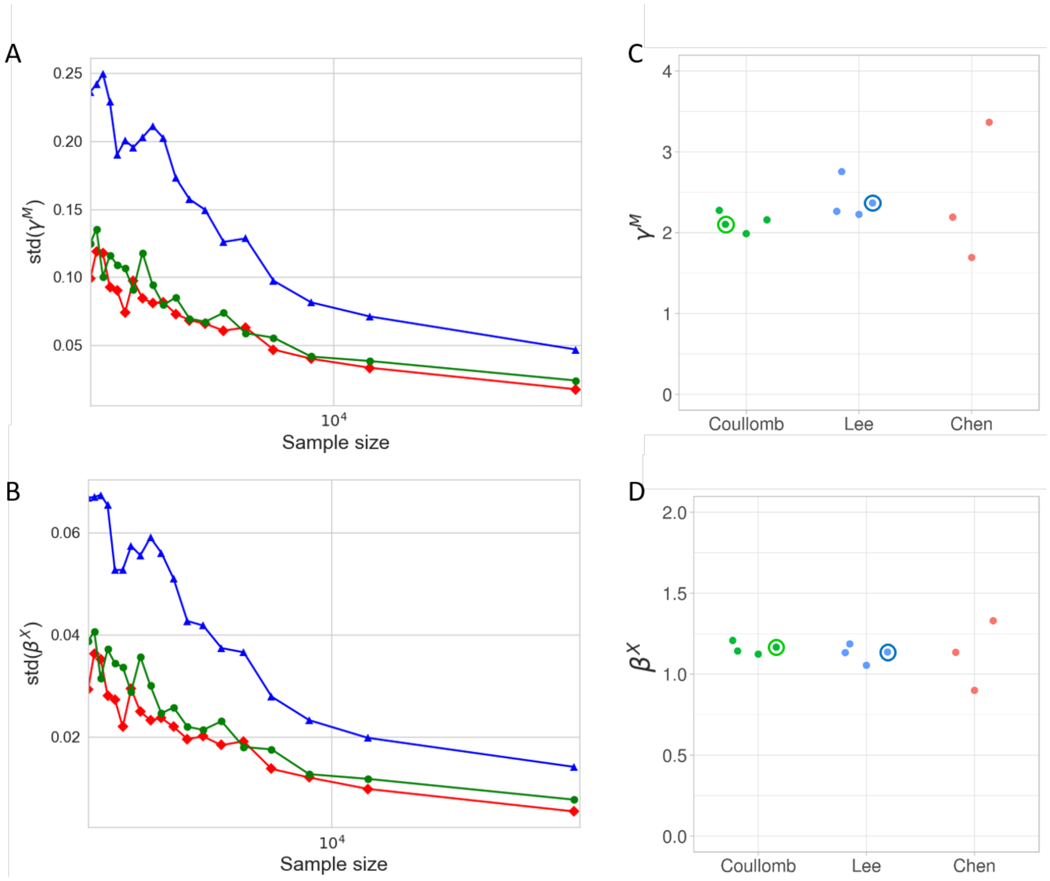

Figure S6: Standard deviation of the correction factors γ^M (A) and β^X (B) obtained by three calibration methods and from a random down-sampling of the experimental dataset. Blue triangles: Lee et al., red diamonds: Chen et al. and green circles: this work. γ^M (C) and β^X (D) obtained with the three methods by reducing the dataset to only utilize two of the three standard constructs. All three possible combinations are shown. The fourth point for Coullomb (QuanTI-FRET) and Lee shown as outlined symbol is the total dataset.

A systematic comparison was performed with two other quantitative FRET methods introduced earlier by Lee et al.[5] and Chen et al.[4]. The comparison focuses on the determination of the two correction factors γ^M and β^X, the crosstalk and direct excitation determinations being well established (α^{BT} and δ^{DE}). The first row of panels in Fig.S6 presents the results for γ^M while the second row shows the results for β^X. A bootstrap analysis was done by chopping the data into smaller datasets to investigate the convergence of the determined correction factors with the sample size. In this test, Chen's and QuanTI-FRET methods performed similarly and better than Lee's method. In the other test, the calibration was achieved by keeping the data from only two FRET standards, three combinations being possible (C5V/C17V, C5V/C32V and C17V/C32V). Here, Lee's and QuanTI-FRET methods performed equivalently while Chen's method seemed more sensitive. All in all, the QuanTI-FRET method appears to be the most robust of the three tested methods.

Comparative table of the analyses of the data from the two independent laboratories

	α^{BT}	δ^{DE}	β^{X}	γ^{M}	C5V	C17V	C32V
lab [A]	0.421 ± 0.002	0.1100 ± 0.0008	1.167 ± 0.008	2.10 ± 0.02	50.3 ± 0.4	41.7 ± 0.8	35.1 ± 0.8
lab [B]	0.467 ± 0.001	0.101 ± 0.003	2.03 ± 0.07	1.35 ± 0.07	50 ± 1.7	43 ± 1.6	33 ± 1.5

Table S1: Comparative table of the correction factors calculated for the analyses for experimental data obtained in laboratories [A] and [B]. Crosstalk correction factors are close and reflect the same fluorophore pair and similar filter sets. The difference between β^{X} and γ^{M} is larger, these factors reflect the relative illmuination intensities and the relative instrumental detection efficiencies in both channels. Interestingly two different cameras were used for the two systems, a sCMOS camera in lab [A] and a EMCCD in lab [B]. After calibration, the FRET probabilities calculated for each FRET standard are in good agreement between lab [A] and [B].

References

1. Thaler, C., Koushik, S. V., Blank, P. S. & Vogel, S. S. Quantitative Multiphoton Spectral Imaging and Its Use for Measuring Resonance Energy Transfer. *Biophys. J.* **89**, 2736–2749, DOI: 10.1529/biophysj.105.061853 (2005).

2. Koushik, S. V., Chen, H., Thaler, C., Iii, H. L. P. & Vogel, S. S. Cerulean , Venus , and VenusY67c FRET Reference Standards. *Biophys. J.* **91**, L99–L101, DOI: 10.1529/biophysj.106.096206 (2006).

3. Koushik, S. V. & Vogel, S. S. Energy migration alters the fluorescence lifetime of Cerulean: implications for fluorescence lifetime imaging Forster resonance energy transfer measurements. *J. Biomed. Opt.* **13**, 031204, DOI: 10.1117/1.2940367 (2008).

4. Chen, H., Puhl, H. L., Koushik, S. V., Vogel, S. S. & Ikeda, S. R. Measurement of FRET Efficiency and Ratio of Donor to Acceptor Concentration in Living Cells. *Biophys. J.* **91**, L39–L41, DOI: 10.1529/biophysj.106.088773 (2006).

5. Lee, N. K. *et al.* Accurate FRET measurements within single diffusing biomolecules using alternating-laser excitation. *Biophys. J.* **88**, 2939–53, DOI: 10.1529/biophysj.104.054114 (2005).
